# Towards the molecular mechanism of the integration of peroxisomal membrane proteins^☆^

**DOI:** 10.1016/j.bbamcr.2015.09.031

**Published:** 2016-05

**Authors:** Evdokia-Anastasia Giannopoulou, Leonidas Emmanouilidis, Michael Sattler, Gabriele Dodt, Matthias Wilmanns

**Affiliations:** a EMBL Hamburg, c/o DESY, Building 25A, Notkestraße 85, 22603 Hamburg, Germany; b Institute of Structural Biology, Helmholtz Zentrum München, 85764 Neuherberg, Germany; c Center for Integrated Protein Science Munich (CIPSM) at Department of Chemistry, Technische Universität München, Lichtenbergstr. 4, 85747 Garching, Germany; d Interfaculty Institute of Biochemistry, University of Tübingen, Hoppe-Seyler-Str. 4, 72076 Tübingen, Germany; e University of Hamburg Clinical Center Hamburg-Eppendorf, Martinistraße 52, 20246 Hamburg, Germany

**Keywords:** Peroxisome biogenesis, Pex3, Pex19, Peroxisomal membrane proteins, Structural biology

## Abstract

The correct topogenesis of peroxisomal membrane proteins is a crucial step for the formation of functioning peroxisomes. Although this process has been widely studied, the exact mechanism with which it occurs has not yet been fully characterized. Nevertheless, it is generally accepted that peroxisomes employ three proteins – Pex3, Pex19 and Pex16 in mammals – for the insertion of peroxisomal membrane proteins into the peroxisomal membrane. Structural biology approaches have been utilized for the elucidation of the mechanistic questions of peroxisome biogenesis, mainly by providing information on the architecture of the proteins significant for this process. This review aims to summarize, compare and put into perspective the structural knowledge that has been generated mainly for Pex3 and Pex19 and their interaction partners in recent years. This article is part of a Special Issue entitled: Peroxisomes edited by Ralf Erdmann.

## Highlights

• Structures of the PMP insertion factors Pex3 and Pex19 and their interactions with other protein ligands
• Structural insights provide a mechanistic understanding of the PMP functional network.
• Functional implications of structural order/disorder transitions

## 1 Introduction

Peroxisomes are cell organelles found in virtually all multi-cellular organisms and comprise a separate cellular compartment whose main functions include the sequestration of metabolic processes that would otherwise be toxic to the cell, as well as the biosynthesis of several essential lipids [1]. The maintenance of the peroxisomal system involves several processes like peroxisome biogenesis, peroxisome growth and division [2], [3], [4] and turnover of peroxisomes by autophagy [5], [6], [7]. One of the most critical steps in peroxisome biogenesis is the correct spatial and temporal topogenesis of peroxisomal membrane proteins (PMPs) on the peroxisomal membrane.

The mechanistic elucidation of the peroxisomal import machinery for the proper integration of PMPs poses major challenges to peroxisomal research. Advanced live and high-resolution imaging techniques have recently allowed the visualization of previously undetected peroxisome biogenesis pathways. However, structural insight into the underlying molecular steps at atomic resolution is still at an early and incomplete stage. This review summarizes recent findings on available structural data of protein complexes and provides structure-based mechanistic interpretation of the processes they are involved in. It will conclude with a range of questions that we believe are critical and should be addressed in future research.

From known peroxisomal biogenesis factors only three – Pex3, Pex19, and Pex16 in mammals – have been shown to be essential for peroxisomal membrane biogenesis [8], [9]. In the absence of any of these, no peroxisomal structures can be identified and PMPs are mistargeted or become degraded [10]. The origin of peroxisomes and their biogenesis is a highly debated research topic, ultimately leading to the fundamental question whether peroxisomes are autonomous organelles, like mitochondria and chloroplasts, or part of endomembrane systems and specifically the endoplasmic reticulum (ER). For many years, the prevailing model assumed the biogenesis of peroxisomes to be driven by growth and dynamin-related fission from pre-existing ones [3].

Early studies in *Yarrowia lipolytica* indeed revealed that the glycosylation pattern of two peroxins originates from the ER [11]. However, it was the genetic and imaging data from the Tabak group and others that demonstrated a Pex19-dependent connection of Pex3 to the ER in yeast [12], [13], [14]. Further indications for ER-based sorting mechanisms on several PMPs and transport via pre-peroxisomal vesicles (ppVs) were provided later on [15], [16].Pros and cons associated with these two models, as well as alternative, refined models, have been widely reviewed and are an ongoing subject of intensive research [10], [17], [18], [19], [20], [21], [22]. A detailed analysis of the arguments is challenging, as it requires comparison of diverging experimental data from different model organisms, mostly yeast and mammalian cells, and is beyond the scope of this review. There is increasing evidence however, that both pathways exist in parallel with different kinetics that may vary under different growth conditions and metabolic requirements [18], [23].

## 2 Peroxisomal membrane proteins (PMPs)

Peroxisomal biogenesis is mainly driven by the formation of the appropriate membrane composition required for the correct function of mature peroxisomes, thus allowing the import of peroxisomal matrix proteins into a sequestered cellular environment via their corresponding import machineries. This includes proteins, lipids and other metabolites found in the peroxisomal membrane. Previous research has mostly focused on the composition of the peroxisomal membrane proteins (PMPs).

Generally, PMPs are synthesized on free polyribosomes in the cytosol and are inserted post-translationally into the peroxisomal membrane [3]. Most PMPs contain one or more non-overlapping signal motifs referred to as Membrane Peroxisome Targeting Signal (mPTS) that consists of a Pex19 targeting element and a membrane-anchoring sequence [24]. PMPs that harbor an mPTS motif have been annotated as class I. In some of them the Pex19 interaction domain and the peroxisome sorting sequence has been reported to be functionally or physically separated [24], [25], [26]. Known mPTS sequences are characterized by the presence of one or more short and usually α-helical segments, comprising of positively charged and hydrophobic residues, in addition to at least one transmembrane segment [24], [25], [27], [28], [29], [30]. The mPTS of some PMPs reportedly contains multiple targeting signals, which may reflect possible sorting to discrete peroxisome populations [31]. Despite these general similarities in mPTS sequences, it has remained difficult to deduce an unambiguous mPTS sequence consensus, partly because structural information concerning mPTS recognition by Pex19 is not available.

A subclass of peroxisomal mPTS-containing PMPs belongs to tail-anchored (TA) proteins, which are integral membrane proteins that contain a short polar C-terminal sequence segment adjacent to a transmembrane domain segment [32]. TA proteins that are directed towards the ER are generally recognized and translocated by the ER-associated and ATP-dependent GET pathway [33], [34]. However, for a number of TA PMPs – including the mammalian Pex26, and Fis1 – it has been recently shown that they can be imported directly to the peroxisomal membrane in a Pex19-dependent way, while yeast PEX15 – the homolog of PEX26 – is imported into the ER in a Get3-dependent topogenesis [35], [36], [37], [38], [39].

There are other PMPs without an identifiable mPTS recognition motif, known as class II PMPs, suggesting Pex19-independent transport and import into the peroxisomal membrane, first discovered and characterized for the peroxisomal docking biogenesis factor Pex3 [40]. Pex3 later on became the paradigm PMP to study the indirect targeting pathway via the ER (further details below). Several other PMPs have also been found to be directly associated with the ER, or structures emerging from the ER, in particular Pex16 in plants and mammals, and Pex15 and Pex22 in yeast [8], [16], [41].

## 3 Peroxisomal biogenesis factor 19 (Pex19)

Pex19 is an essential, multifunctional peroxisomal protein component that is ubiquitously found in peroxisome-containing organisms. It plays central but functionally different roles in the established models of peroxisome biogenesis [9], [18], [21], [42]. When considering the growth and division model, Pex19 binds and stabilizes newly synthesized PMPs via their mPTS recognition motif, thus also acting as a quality control component for proper folding, before delivering them to the peroxisomal membrane in a Pex3-dependent manner [35], [40]. Due to these findings, Pex19 has been ascribed a role as both shuttling PMP receptor and chaperone, with the ability of self-recovery after PMP release.

In the de novo peroxisomal biogenesis model the proposed key role of Pex19 is ATP-dependent budding of ppVs from the ER, which subsequently convert to mature peroxisomes [18], [19]. Less clear is a suggested role of Pex19 in possibly regulating the docking process of cargo-loaded Pex5 matrix protein receptor, by binding to Pex14 [20], [43]. Recent evidence has also indicated a role of yeast Pex19 in peroxisomal inheritance by binding directly to the myosin motor protein Myo2 in a Pex19-farnesylation dependent manner [44]. The precise Myo2 binding site on Pex19, however, remains unknown.

**Fig. 1:**
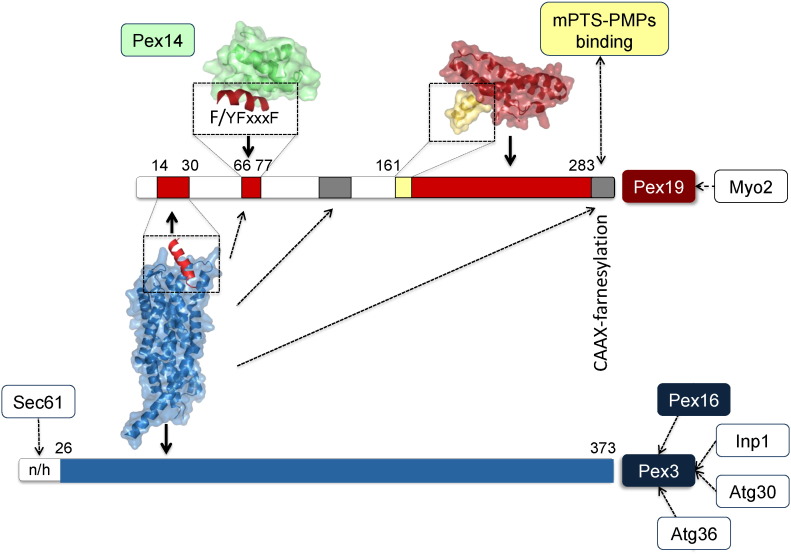
Molecular structures of peroxisomal biogenesis factors and complexes. Available high-resolution structures are shown with semitransparent surfaces and underlying ribbons. Residue numbers of the boundaries of known structures are shown. Protein/protein interactions, which have been confirmed structurally, are indicated with solid arrows; other reported interactions are indicated with dashed arrows. Reported protein interactions, which have not yet been mapped (including the *Saccharomyces cerevisiae* proteins Sec61, Inp1, Atg30, Atg36, Myo2 and with human Pex16), are indicated schematically. For further details, see text. Protein models were made using Pymol [80].

Among vertebrates, Pex19 sequences are highly conserved and most of them comprise 299 residues in length. In contrast, Pex19 sequences from plants, fungi and amoebozoa are diverse in sequence and length, suggesting functional diversity. The C-termini of Pex19 sequences are defined by a so-called CAAX motif, which serves as a recognition site for farnesylation. Virtually all annotated Pex19 sequences harbor this motif, with the trypanosomal Pex19 being an exception [45]. Experimentally, Pex19 farnesylation in vivo has only been demonstrated for a limited number of systems, such as human cell lines and yeast [9]. In contrast, BLAST sequence homology searches [46] reveal that some Pex19 sequences, even closely related to mammalian Pex19 (PEX19_HUMAN), comprise N-terminal extensions of several hundreds of residues. However, to the best of our knowledge none of these extended sequences has been investigated in detail, to date. Basically, Pex19 can be divided into two parts, a structurally flexible N-terminal region and a structured C-terminal region, which includes the C-terminal CAAX motif (Fig. 1). The C-terminal region of Pex19 sequences is generally more conserved than the N-terminal part, with the exception of residues 17–23 (human Pex19 residue numbering) that include several highly conserved residue positions as well.

Full-length Pex19 is monomeric, as determined by analytical ultracentrifugation, but under gel filtration conditions it elutes with an unusually high apparent molecular weight [47], [48], [49]. This finding has been attributed to its predicted shape as a partly non-globular protein. It could also explain data on Pex19 from *Arabidopsis*, which has been interpreted as an indication for a dimeric arrangement [50]. The helical content of Pex19 was estimated to be 6% for the N-terminal part and 55% for the C-terminal part [47]. Pex19 is highly soluble and can be purified to concentrations exceeding 100 mg/ml [47].

The three-dimensional structure of full-length Pex19 remains unknown, although structural insights in domain fragments have become available (Fig. 1). The structure of most of the globular C-terminal domain of human Pex19, lacking the CAAX box sequence motif (residues 161–283), has been determined by X-ray crystallography (PDB code 2WL8) and can be described as a largely α-helical bundle domain [51]. For the less folded N-terminal part of Pex19, structures of short peptide stretches of human Pex19 comprising residues 15–40 (PDB code 3AJB) and 14–30 (PDB code 3MK4) have been determined in complex with the docking factor Pex3 (details below) [51], [52]. Taking the data together, residues 14–32 are presumed to be in an α-helical conformation in the presence of Pex3, whereas this segment is unstructured in the absence of Pex3 [49], [52]. Residues 66–77 of human Pex19 form an α-helix when bound to Pex14 (details below, PDB code 2W85, Fig. 1), as determined by NMR spectroscopy [53]. A number of additional segments within the N-terminal region of Pex19 have been predicted to be folded in amphipathic α-helices and have been implicated in PMP membrane insertion [39].

Pex19 has multiple protein/protein interaction regions (Fig. 1). Probably the most thoroughly investigated one is with the membrane docking protein Pex3, the binding affinity of which has been determined to be ≤ 10 nM [52], [54]. The high affinity of this complex seems to be caused by an intricate set of interactions, exceeding a simple binary binding site. The affinity of the N-terminal binding site that has been determined structurally (further details described below) is about 5–10 less than that for full-length Pex3, but sufficient to establish a stable and constitutive protein/protein complex [52], [54]. The finding of a second low affinity Pex3 binding site within the central part of Pex19 (residues 124–140) [55], [56] could explain the difference in binding affinity but does not appear to be sufficient to establish binding on its own [54], [57]. In a recent study, another sequence stretch in the N-terminal part of Pex19 (residue 64–74) and the C-terminus of Pex19 comprising the CAAX box for farnesylation have been found to be affected by Pex3 binding [49]. Human PMP24 cargo-loaded Pex19 displays increased binding affinity to Pex3, [58] suggesting that conformational changes are involved in regulating the affinity of Pex3/Pex19 assembly.

In addition to the Pex3 binding site, the N-terminal part of Pex19 contains a Pex14 interacting region. Pex14 is a peroxisomal membrane protein that is an essential component of the peroxisomal matrix protein import machinery. Contrary to other PMPs, Pex14 does not contain a typical mPTS motif. Whether the targeting and insertion of Pex14 is Pex19-dependent or more relying on the Pex13 interaction is not yet fully understood [59], [60]. Recent data show that Pex14, together with other peroxisomal importomer protein components for peroxisomal matrix protein import – Pex8 and Pex13 – are found in ppVs in *Saccharomyces cerevisiae* [20], [43]. What kind of role the Pex14/Pex19 interaction could play in the same subsequent maturation into import-competent peroxisomes, however, still remains to be determined.

**Fig. 2:**
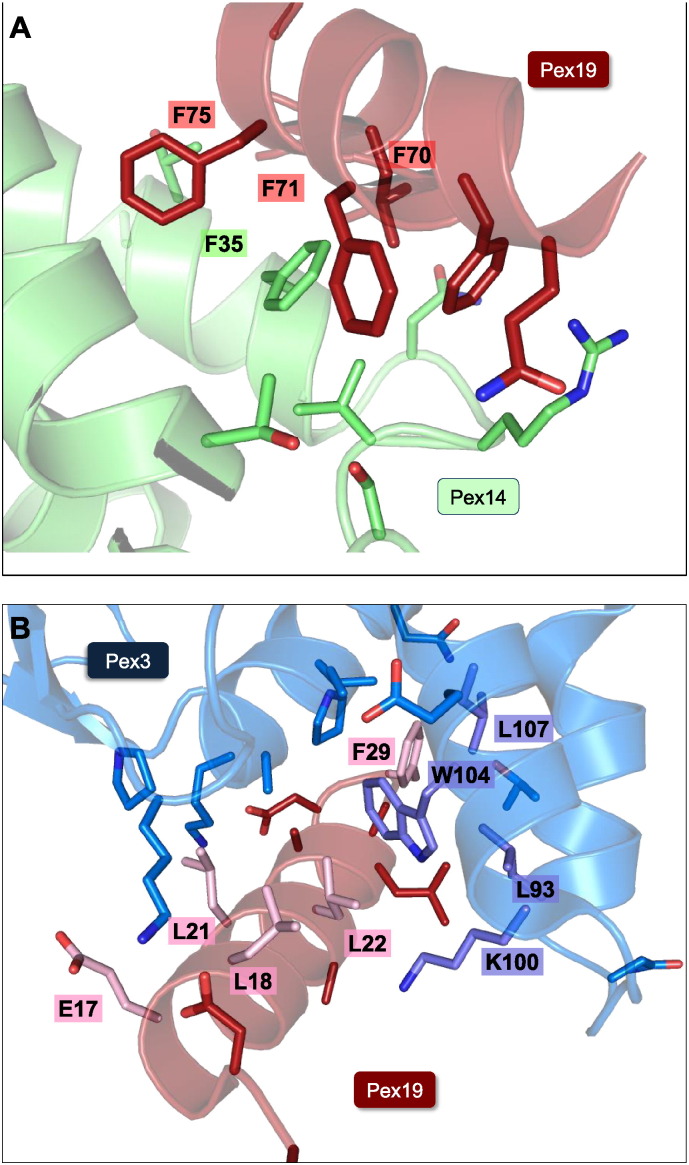
Protein/protein interfaces of the complexes involving peroxisomal biogenesis factors. The side chains of residues with contributions with > 10 Å^2^ accessible surface buried upon complex formation, as calculated with PISA [81], are shown. (A) Pex19–Pex14: none of the residues involved in the respective interface are universally conserved by the criteria given above. Aromatic residues from both Pex14 and Pex19 that establish the staggered array of aromatic residues in the interface are labeled. (B) Pex3–Pex19: Residues, which are universally conserved according those found with a BLAST search using the *H. sapiens* sequence in UNIPROT as template and those reviewed in Swiss-Prot selected for an overall alignment, are highlighted in bold colors and labeled.

The interaction between Pex19 and Pex14 has been investigated by NMR spectroscopy, and solution structures of the N-terminal α-helical domain (NTD) of Pex14 and a peptide comprising Pex19 residues 66–77 (PDB code 2W85) have been reported. The recognition of Pex19 peptide depends on a F/YFxxxF sequence motif (Fig. 1) [53]. In the same study, the structure of the Pex14 NTD bound to a WxxxF/Y sequence motif of the peroxisomal matrix protein import receptor Pex5 was also presented. Notably, both peptide motifs bind in a helical conformation to the same site in the Pex14 NTD, although with different affinities. The dissociation constants for the interaction with the Pex5 and Pex19 motifs are 0.5 and 9 μM, respectively. The Pex5/Pex19 binding domain of Pex14 forms two hydrophobic cavities that recognize the two flanking aromatic side chains from either the Pex5 WxxxF/Y motif or the Pex19 F/YFxxxF motif (Fig. 2A). Both the Pex5 and Pex19 peptides form an amphipathic helix that binds across two helices of the N-terminal Pex14 helical bundle domain. Surprisingly, the two Pex14-binding motifs of Pex5 and Pex19 are in opposite orientations. It is also interesting to note that the same Pex19 F/YFxxxF sequence motif is affected by Pex3 binding (see above) [49], suggesting a possible competition of Pex3 and Pex14 for the same Pex19 site. However, this still requires experimental verification.

Only little is known on the mechanism of mPTS-mediated binding by PMPs to Pex19. PMP binding data from truncated Pex19 constructs indicated a crucial contribution of the first visible helix (residues 171–182) in the C-terminal Pex19 domain (Pex19 CTD) structure to PMP binding [51]. The binding affinity corresponds to a dissociation constant *K*_D_ of about 10 μM and is thus moderate [51]. Replacement of a number of exposed hydrophobic residues from this helix leads to loss of mPTS binding, indicating non-specific hydrophobic interactions to be crucial. In the same study it was shown that the presence of the non-farnesylated CAAX motif considerably reduces the binding affinity [51]. In independent experiments it was demonstrated that CAAX-mediated farnesylation increases the binding tenfold [61], suggesting opposite regulatory roles of the C-terminal CAAX motif depending on whether it is farnesylated.

The *apo*-structure of the Pex19 CTD reveals a large cavity in the core of the domain, which has been suggested to function as an intermolecular binding site of the farnesyl group [51] (Fig. 1). A comparison of NMR fingerprint spectra of the non-farnesylated and farnesylated Pex19 CTD is consistent with a burial of the farnesyl group in this cavity (Schütz and Sattler, unpublished). This could explain previous findings of an increased mPTS binding affinity and functional activity of farnesylated Pex19 [61]. The impact of farnesylation for Pex19 function, as judged from in vivo experiments in yeast and mammalian cell lines, however, remains controversial [9]. Remarkably, Pex19 has the ability to actively bind newly-synthesized PMPs as shown by a study of Pex19 with an engineered nuclear localization signal that resulted in enrichment of PMPs in the nucleus [40]. This argues in favor of a stable and constitutive interaction of Pex19 and PMPs. Recent data indicate that the balance of hydrophobic and charged residues in the mPTS of tail-anchored proteins, a PMP subclass, are critical to determine competitive binding to Pex19 and endoplasmic reticulum associated cytosolic ATPase TRC40 (Get3 in yeast) [38]. How PMP cargo-bound Pex19 increases its binding affinity to Pex3 [58] is not yet understood mechanistically.

Considering Pex19 as a shuttle PMP receptor, there is also the question about a possible PMP release mechanism from Pex19. One of the most well-characterized Pex19–PMP complexes is that of Pex26 (Pex15 in yeast) that has also been isolated as ternary complex in the presence of Pex3 [38], [56]. A region in the N-terminal part of Pex19 with predicted amphipathic helical properties has been recently implicated in Pex26 insertion into the peroxisomal membrane by a release mechanism from Pex19 [39]. This segment has been suggested to compete with the mPTS-binding helix from the Pex19 C-terminal domain [51].

## 4 Peroxisomal biogenesis docking factors 3 (Pex3) and 16 (Pex16)

Pex3 is an integral membrane protein that binds to an N-terminal Pex19 site that is different from the Pex19 PMP binding site. Based on this, Pex3 has been proposed to function as Pex19-dependent receptor and membrane docking factor [62]. Unlike many other PMPs, its membrane insertion does not depend on Pex19, categorizing Pex3 as a class II PMP [40]. Similar to Pex19, vertebrate Pex3 sequences are highly conserved and are 372 or 373 residues in length. In contrast, Pex3 sequences from more distantly related organisms such as fungi and plants generally share < 35% sequence identity with human Pex3 and are divergent in length.

Pex3 contains an N-terminal transmembrane segment preceded by a short but distinct basic luminal segment, recently termed n-region and h-regions, due to its similarity with ER signal anchor-like sequences [63]. This segment has also been referred to as mPTS2 [10] and it anchors Pex3 into the peroxisomal membrane and other peroxisomal precursor structures such as the ER and ppVs [63], [64]. Recent data have revealed that the recognition of these two regions in Pex3 is probably dependent on the Sec61 endomembrane channel system associated with the ER [63], providing a mechanistic rational on findings on Pex3 transport via the ER in *S. cerevisiae* [12], [13], [14]. In vertebrates, the ER receptor function for Pex3 is provided by Pex16, which is not found in various yeast species [65]. In contrast to Sec61, Pex16 also serves as a Pex3/Pex19 membrane docking component in mature peroxisomes [66]. The mechanisms of Pex16 membrane insertion itself, however, appears to be different, as it is depends on Pex19 for integration into the mature peroxisome membrane but not for ER integration. Pex16, in which the N-terminal mPTS is deleted, integrates into the ER membrane but not into the membrane of mature peroxisomes [8]. Studies of human Pex16, and the equivalent proteins from *Arabidopsis thaliana*, and *Y. lipolytica*, have revealed substantial functional diversity, which is also reflected in sequence diversity [65]. There is no structural information on Pex16 available, to date.

**Fig. 3:**
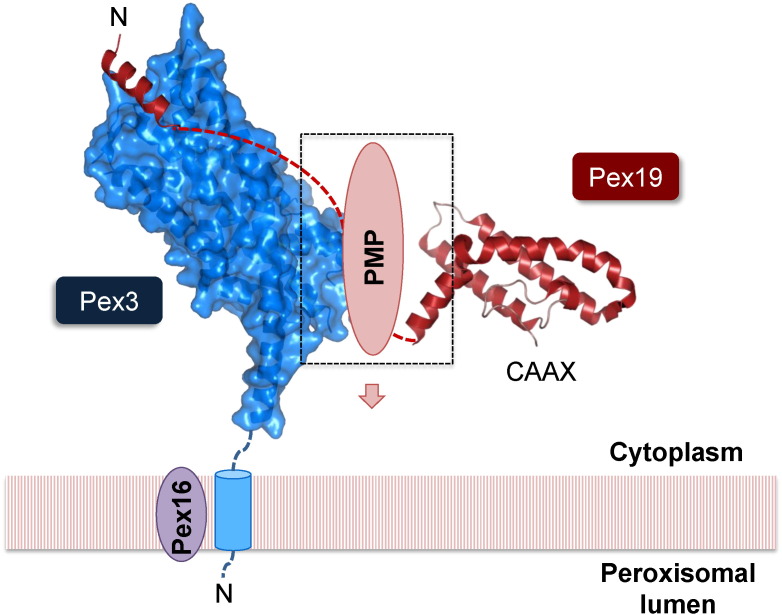
Model of the ternary Pex3–Pex19–PMP complex conceived from known structural and interaction data, as inspired by the model proposed by Schmidt et al. [57]

The remaining soluble part of Pex3 comprises a helical bundle domain, for which the molecular structure has been determined at high resolution by X-ray crystallography in complex with an N-terminal Pex19 peptide fragment (PDB codes 3AJB and 3MK4) [52], [54] (Fig. 1, Fig. 2B). The Pex19 binding site is distally located with respect to the anticipated N-terminal transmembrane anchor. The Pex19 binding site is about 600 Å^2^ in size and thus quite compact. Three Pex3 loop regions contribute to this Pex19 binding site, including the sequence segments 90–107, 196–197 and 321–330. Several residues contributing to the Pex3–Pex19 interaction both in Pex3 and Pex19 are universally conserved among the respective sequences from various species, indicating that the interaction is conserved as well (Fig. 2B). Although a number of specific hydrogen bond interactions contribute to the interaction, remarkably mainly hydrophobic residues involved in binding are conserved both in Pex3 and Pex19. A cluster of conserved hydrophobic residues near the N-terminal membrane insertion site seems to play a role in facilitating PMP membrane insertion by possibly deforming the peroxisomal membrane [39], [57] (Fig. 3). The presence of Pex19 has a limited stabilizing effect on Pex3 in vitro and in cellular assays, but a Pex3 chaperone function of Pex19 has not been detected, unlike for other PMPs [57], [66].

Recent data show new additional links of Pex3 to ubiquitination-linked pexophagy [67], [68], [69], [70], balancing peroxisome biogenesis and maintenance by regulated turnover. Interactions of Pex3 with protein components involved in peroxisomal autophagy, such as Atg36 [71] and Atg30 [72], have been identified, but the precise binding sites on Pex3 and the mechanism of interaction remain unknown. Another interesting Pex3 interaction has been established with the peroxisomal inheritance factor Inp1 [73], [74], which has, likewise, not yet been mapped to a specific Pex3 site. Inp1 has been shown to regulate peroxisome motility, as a requirement for peroxisomal proliferation, and abundance.

## 5 Emerging structural and mechanistic principles

Although the presently available structural data of peroxisomal biogenesis factors and complexes are still very limited, they provide an idea on underlying principles of the formation of relevant protein/protein complexes. The structures of all protein domains from Pex3, Pex14 and Pex19 investigated to date, are exclusively helical, leading to helix/helix interactions observed in respective protein/protein complexes [51], [52], [53], [54] (Fig. 1, Fig. 2). As shown paradigmatically for the peroxisomal matrix protein import receptor Pex5, helical bundle structures have superior properties for conformational adaptations of the overall fold in protein/protein binding events [75], [76].

Some of the helices from peroxisomal biogenesis factors involved in protein/protein interactions comprise a large proportion of conserved hydrophobic surface residues. Assuming that aliphatic helices have normally hydrophobic residues oriented towards the hydrophobic core in globular proteins, in these peroxisomal proteins some of the aliphatic helical properties appear to be inverted, in other words having crucial hydrophobic residues situated on the surface rather than being oriented into the protein interior. Recently it has been shown at a molecular level that these properties are not only meaningful in establishing protein/protein interactions but also in the release of protein ligands, as demonstrated for the disassembly of Pex26 from Pex19/Pex3 within the peroxisomal membrane [39]. Such structural features are found in particular in proteins that are undergoing transitions between lipid-free or lipid-bound states [77], which fit the idea of Pex19 functioning as a shuttling receptor. Interestingly, the role of aliphatic helices, as shown for Pex11, may also have a prominent role in peroxisomal membrane remodeling in the peroxisomal fission process, which complements the de novo biogenesis of peroxisomes [4], [78].

For the N-terminal helical Pex13 interaction site on Pex19 it has been shown that Pex19 folding is induced by Pex13 binding [52]. Although part of the suspected mPTS binding site is folded in the Pex19 C-terminal domain structure possibly because of contacts with neighboring molecules in the crystal lattice [51], it may well be that there are folding transitions in Pex19 upon mPTS binding as well. The observed regulatory role of the farnesylated C-terminal CAAX motif in mPTS supports this hypothesis [51], [61]. It will be of particular interest to investigate to what extent these principles, presently limited to only a few examples, could be found in related systems as well, allowing a broader generalization.

## 6 Open questions and future challenges

The central role of the Pex3/Pex19 complex in peroxisomal biogenesis is well established and, therefore, it can be expected that this complex will be a starting point for future investigations focusing on the underlying molecular structures and associated dynamics. However, although structures of fragments of the Pex3/Pex19 complex have been determined as discussed above, little is known about the architecture and conformational transitions this complex may undergo during the mPTS-PMP delivery process into the peroxisomal membrane. As the mPTS-PMP docking process most likely takes initially place at the membrane surface, as suggested by the structural organization of Pex3 with a separate N-terminal transmembrane segment, the soluble part of the Pex3 in the presence of Pex19 may be sufficient as model system to study mPTS-PMP recognition. Ultimately, future structural investigations need, however, to be confirmed in a native membrane environment. Since many, if not all of these processes, start at the ER and subsequent pre-peroxisomal vesicle structures [15], [16], the respective membrane environments need to be tested. As there is strong evidence from the relevant literature of a high level of diversity in the mechanistic details of peroxisomal biogenesis [79], as a prerequisite to understand those differences, it would be desirable to gain future data for protein complexes from different representative organisms, including higher vertebrates, yeast and plants. There may be also important roles of organism-specific post-translational covalent modifications, which have not been widely addressed in previous research. Key future research questions we suggest to be addressed could be:

• How does mPTS-binding occur and how does Pex19 act as a chaperone? Are there specific interactions involved? What is the distributed role of hydrophobic and charged residues found in mPTS sequence motifs? What kind of conformational PMP transitions are found in the presence of Pex19?
• What kind of conformational transitions are found in the Pex3/Pex19 complex during its formation, all stages of mPTS-PMP binding and release, and Pex3/Pex19 disassembly? What is the mechanism of disassembly of the Pex3/Pex19 complex, whose affinity has been measured in the nM range? What is the role of previously reported additional secondary Pex3/Pex19 binding sites? What is the contribution of Pex19 farnesylation in the overall process?
• What is the mechanism of Pex3/Pex19 mediated mPTS-PMP docking to full integration into the membrane? What is the role of Pex16, the known ER GET receptors and the Sec61 translocon?

As these complexes comprise significant unfolded regions, as exemplified for the N-terminal part of Pex19, it will be challenging to capture the overall architecture of peroxisome biogenesis complexes by X-ray crystallography. Thus, the use of complementary structural biology methods, such as Small Angle X-ray scattering, including options for time-resolved studies, and various biophysical methods, is expected to be essential in future research. NMR spectroscopy has proven to be very useful to map protein interactions and characterize conformational flexibility at the amino acid residue level and thus is expected to play a key role as well. In essence, the use of structural biology for the understanding of peroxisome biogenesis is still very much in its infancy and it will require novel, unconventional approaches to tackle this central challenge in peroxisomal research.
